# Hyaluronic acid-engineered milk extracellular vesicles to target triple negative breast cancer through CD44

**DOI:** 10.1080/13880209.2025.2511807

**Published:** 2025-06-05

**Authors:** Filipa A. Soares, Beatriz Salinas, Salette Reis, Cláudia Nunes

**Affiliations:** ^a^ LAQV, REQUIMTE, Departamento de Ciências Químicas, Universidade Do Porto, Porto, Portugal; ^b^ ICBAS - Instituto de Ciências Biomédicas Abel Salazar, Universidade Do Porto, Porto, Portugal; ^c^ Departamento de Bioingeniería, Universidad Carlos III de Madrid, Madrid, Spain; ^d^ Unidad de Medicina y Cirugía Experimental, Instituto de Investigación Sanitaria Hospital Gregorio Marañón (IiSGM), Madrid, Spain; ^e^ CIBER de Salud Mental, Instituto de Salud Carlos III, Madrid, Spain; ^f^ Unidad de Imagen Avanzada, Centro Nacional de Investigaciones Cardiovasculares (CNIC) Carlos III, Madrid, Spain

**Keywords:** Bovine milk, small extracellular vesicles, hyaluronic acid, breast cancer, selective targeting, CD44

## Abstract

**Context:**

Cancer therapy remains a challenge in healthcare, particularly in the context of triple-negative breast cancer (TNBC), where targeted therapies are still scarce.

**Objective:**

Addressing this issue, our study explores a novel targeting approach using small extracellular vesicles (sEVs) isolated from cow milk, functionalized with hyaluronic acid (HA) to target the overexpressed cluster of differentiation 44 (CD44) cell surface receptor in TNBC cells.

**Materials & methods:**

A method for isolating sEVs from cow milk was optimized, and the obtained sEVs were fully characterized in terms of size, morphology, and protein markers. Subsequently, milk-derived sEVs were covalently bound with HA of varying molecular weights (MW, 20–60 kDa, 250 kDa, 1000–1600 kDa) and binding and internalization dynamics were investigated. Breast cancer cell lines, MDA-MB-231 (TNBC and CD44+) and MCF-7 (CD44-), were used as *in vitro* models to evaluate CD44 selectivity.

**Results:**

The binding and internalization studies unveiled enhanced selectivity of functionalized sEVs for CD44-overexpressing cells compared to non-functionalized sEVs. Notably, higher MW HA exhibited enhanced binding capacity, with partial internalization occurring through CD44 endocytic mechanisms.

**Discussion and conclusion:**

In summary, this work introduces a sEVs isolation method and sheds light on the role of HA MW in enhancing cellular uptake of CD44 overexpressing cancer cells.

## Introduction

Cancer is still one of the most life-threatening diseases of our days. Specifically, triple negative breast cancer (TNBC) is the breast carcinoma subtype that presents the worst clinical outcomes (Jain et al. 2020). Presently, TNBC treatment predominantly revolves around surgical interventions, radiation therapy, and chemotherapy. This type of treatment, specifically chemotherapy, causes severe off-target side effects, with associated low efficacy, due to reduced bioavailability of the chemotherapeutic agents and the risk of resistance development (Mendes et al. 2015; Jain et al. 2020). Such hurdles have prompted the involvement of nanotechnology in the cancer medicine field, by providing the tools to achieve targeted drug delivery (Mendes et al. 2015). Additionally, the high degree of heterogeneity characterizing TNBC tumors has posed great challenges in the development of targeted therapies. Despite recent process, including the introduction of PARP inhibition, immunotherapy employing checkpoint inhibitors, or the utilization of topoisomerase inhibitors, success remains limited (Chapdelaine and Sun 2023). Just around 20% of TNBC patients typically exhibit genomic eligibility for these therapies, with positive outcomes for only half of them (Marquart et al. 2018). This lack of broadly effective targeted therapy for TNBC shows the urgent need for novel strategies to address this pressing medical challenge. In this context, cluster of differentiation 44 (CD44) has been receiving much attention. It is a cell surface glycoprotein that is overexpressed in different solid tumors, including TNBC tumors (Paulis et al. 2015; Cadete and Alonso 2016). In fact, high expression of CD44 has been associated with increased tumour aggressiveness, by promoting tumor cell plasticity and chemoresistance (Paulis et al. 2015; Zhao et al. 2016). Additionally, CD44 is also considered a surface marker for cancer stem cells (Guo et al. 2022). This makes CD44 one of the most promising biomarkers for cancer diagnosis, poor prognosis, as well as therapeutic targeting for highly aggressive cancers. Hyaluronic acid (HA) is a natural polysaccharide that is a native ligand of CD44 (Cadete and Alonso 2016). It has been demonstrated, however, that the interaction between CD44 and HA is highly dependent on the activation state of the receptor (Liu et al. 1998; Moll et al. 1998; Yang et al. 2015). CD44 is endogenously expressed in both normal and tumor cells (Yang et al. 2015). Nonetheless, in normal cells, this receptor, despite featuring the amino-terminal HA-binding motif, are resting under physiological conditions, not capable of binding HA. In contrast, due to the inflammatory nature of the tumor microenvironment, CD44 is in an active state and can bind and internalize HA (Guo et al. 2022). This allows a high degree of selectivity in targeting CD44 on tumor cells. Therefore, HA has been thoroughly investigated to actively targeting tumors using several types of nanoparticles (micelles, liposomes, lipid-based, inorganic nanoparticles) (Fu et al. 2023).

Recently, a new generation of biomimetic nanoparticles, namely small extracellular vesicles (sEVs), has been emerging as natural alternatives to the so-called conventional nanoparticles (Liu et al. 2023). sEVs are lipid bilayer vesicles that are naturally secreted by cells and have been proposed as a promising alternative to synthetic liposomes (González et al. 2023). There vesicles, also called ‘exosomes’, have an endosomal origin and play a role in cell-to-cell communication due to their bioactive cargo (lipids, metabolites and RNA) (González et al. 2023). In the use as drug delivery systems, their nanometric size (between 30 and 150 nm) and lipid bilayer structure represent major advantages (Soares et al. 2023). sEVs can be loaded with both hydrophilic and hydrophobic molecules and may present longer circulation times in the bloodstream compared to synthetic liposomes (González et al. 2023; Soares et al. 2023). These natural vesicles are found in various biological fluids and milk, especially bovine milk, has attracted our interest. Due to its ease of accessibility, large production volumes, and higher isolation yield compared to cell culture or other biological fluids, it becomes a scalable source of sEVs (Adriano et al. 2021). In addition, sEVs from cow’s milk are considered biocompatible and have been shown to be tolerated across species. They have also shown positive modulating effects in terms of inflammation and protective effects against oxidative stress on the intestinal mucosa (Wang et al. 2021; Mecocci et al. 2022).

With this in mind, this work aimed to study the potential of HA-engineered sEVs to target CD44 overexpressing TNBC cells. Envisioning a future application in cancer therapy but also for diagnostic and prognosis purposes, the sEVs were also fluorescently labeled to allow the detection of the targeted cells. An isolation and purification method of sEVs from raw cow milk was optimized, and the obtained sEVs were fully characterized. sEVs were fluorescently labeled and then functionalized with HA, through covalent binding to the surface proteins of the sEVs. Despite other receptors are able to bind HA, such as RHAMM and ICAM-1 (Marinho et al. 2021) the CD44 overexpression in TNBC is notably high, thus, it is expected that the accumulation of HA-functionalized sEVs will be significantly greater in CD44-overexpressing tumors. Being the molecular weight (MW) of HA another recognized variable capable of influencing its binding affinity with the receptor (Guo et al. 2022), herein we also analyzed the binding and internalization of three different HA MW as targeting moieties.

## Materials and methods

### Isolation of sEVs from bovine milk

sEVs were isolated from raw bovine milk obtained from Vairão Agricultural Campus of School of Medicine and Biomedical Sciences. To remove the cream layer and cell debris, fresh milk samples were centrifuged at 14,000 x g, at 4 °C, for 30 min, in 50 mL PPCO tubes (Fisher Scientific^TM^, #10614321, Thermo Fisher Scientific, MA, USA), in a Sorvall LYNX 6000 Superspeed centrifuge with a T29-8 × 50 Fixed Angle Rotor (Thermo Fisher Scientific, #75006590 MA, USA). The skimmed milk was then stored at −80 °C until use. Prior to the isolation of sEVs, skimmed milk was thawed at 37 °C, followed by centrifugation at 5000 x g, for 10 min, at 4 °C, to remove remaining fat globules and cell debris. The supernatant was warmed up until 37 °C and treated with microbial rennet (2.5%(v/v), Browin, #411201, Łódź, Poland), for approximately 30 min, to allow casein coagulation according to previous protocols (González et al. 2023). The casein was removed afterwards by centrifugation at 5000 x g, for 10 min, at 4 °C. The resultant milk whey was subjected to sequential centrifugation at 13,000 x g (35 min, 4 °C), 35,000 x g (45 min, 4 °C) and 70,000 x g (35 min, 4 °C) to exclude contaminant proteins and large vesicles. The sEVs were precipitated by ultracentrifugation of the resultant cleaned whey at 100,000 x g for 90 min, at 4 °C. The resultant pellet was washed twice and resuspended in phosphate-buffered saline (PBS). Additionally, a qEV2 35 nm size exclusion chromatography column (IZON Science, #IC2-35 Lyon, France) was used to further purify the sEVs suspension. Fractions enriched in sEVs were concentrated by ultracentrifugation at 100,000 x g for 90 min, at 4 °C. The pellet was again resuspended in Dulbecco’s Phosphate Buffered Saline 10X (PBS, #D8537, Sigma-Aldrich^®^, St Louis, MO, USA) and stored at −20 °C until use.

### Protein content determination

The protein content of the sEVs suspensions was quantified by the bicinchoninic acid assay, using Pierce^™^ BCA Protein Assay Kit (#23225, Thermo Fisher Scientific, MA, USA). The assay was performed according to the manufacturer’s instructions. Briefly, 25 µL of each sample or standard (20–2000 µg/mL) was pipetted into a 96-well microplate, followed by the addition of 200 µL of working reagent. Protected from light, the plate was incubated at 37 °C for 30 min, and equilibrated to room temperature before measuring absorbance at 562 nm. A standard curve was generated using bovine serum albumin (BSA, provided with the kit), and sample concentrations were calculated by blank-corrected interpolation. The absorbance was measured with a Cytation 3 (Model CYT3MF, BioTek Instruments Inc., Winooski, VT, USA).

### Activation of HA

Hyaluronic acid (Mw 250 kDa) was a kind gift from Bloomage Biotechnology (Jinan, P.R. China). Hyaluronic acid (Mw 20–60 and 1400–1800 kDa) was a kind gift from Freshine Chemicals Company (Parsippany, NJ, USA). 100 mg of HA of different MW (20–60 kDa, 250 kDa, 1000–1600 kDa) were dissolved in 25 mL of ultrapure water, followed by incubation with 0.5 g of ethyldimethylaminopropyl-carbodiimide (EDC, #39391, Sigma-Aldrich^®^, St Louis, MO, USA) and 0.52 g of N-hydroxysuccinimide (NHS, #130672, Sigma-Aldrich^®^, St Louis, MO, USA) at pH 4.0 for 2 h at 37 °C. The mixture was then dialyzed against 1 L phosphate buffer for 48 h, using a cellulose dialysis bag (Cellu.Sep^®^ T1 with a nominal MW cutoff of 3.5 kDa, #11425859, Fisher Scientific^TM^, Thermo Fisher Scientific, MA, USA). The purified HA NHS ester was then lyophilized using a LyoQuest 85 plus v.407 Telstar freeze dryer (Telstar^®^ Life Science Solutions, Terrassa, Spain), yielding a white dry powder. The characterization of the synthethized HA-NHS-ester was performed by Fourier-Transform Infrared Spectroscopy. The spectra of HA and HA-NHS-ester were obtained using a FrontierTM FTIR (Perkin Elmer, Santa Clara, CA, USA) equipped with an attenuated total reflectance (ATR) device and zinc selenite crystals. The results were obtained by combining 16 scans and the spectra were recorded between 4000 and 600 cm^−1^ with a resolution of 4 cm^−1^.

### sEVs labelling and functionalization with HA

Optical labelling of sEVs was adapted from (González et al. 2021). A suspension of 75 µg of sEVs protein in 100 µl was mixed with 1 µL of commercial Sulfo-Cyanine 5 NHS ester (#23320, Lumiprobe, Hannover, Germany) in a concentration of 17 mM. The pH was adjusted to 8.5 (with NaHCO_3_ 0.1 M, prepared from powder sodium bicarbonate (#S233, Fisher Scientific^TM^, Thermo Fisher Scientific, MA, USA) and the mixture was left under agitation at 4 °C. After 4h, the volume was adjusted to 2 mL and placed in cellulose dialysis bags with a 3.5 kDa MW cut-off (Cellu.Sep^®^ T1), sealed at both ends. The dialysis was performed for 24h, in PBS at 4 °C. After the fluorescence labelling, the sEVs were functionalized with HA of different MWs (20–60 kDa, 250 kDa, 1000–1600 kDa). A ratio of 1:5 ratio (v/v) of HA-NHS-ester (10 mg/mL) and sEVs-SCy5 (37.5 µg/mL) were incubated, at pH 8.5, during 4h under agitation.

### Physicochemical characterization

#### Nanoparticle tracking analysis

The hydrodynamic sizes of control and functionalized sEVs were measured using nanoparticle tracking analysis (NTA, NanoSight NS300, Malvern Instruments, Worcestershire, UK) equipped with a 488 nm laser and a high-sensitivity scientific CMOS camera. Samples were injected using a 1 mL sterile syringe in the viewing chamber and analyzed under constant flow (infusion rate of 40). Size measurements were obtained based on 3 different 60s videos captured with a screen gain of 1.0, a camera level of 14 and a detection threshold of 10. Data were analyzed using the NTA 3.4. software.

#### Transmission electron microscopy

A morphological analysis of sEVs was performed by Transmission electron microscopy (TEM) using a JEOL JEM-1010 from ICTS Centro Nacional de Microscopía Electrónica (Universidad Complutense de Madrid, Spain), which operates at 100 kV. Formvar carbon coated copper grids were employed for the negative staining of sEVs with uranyl acetate at room temperature.

#### Western blot

The presence of the sEVs protein markers TSG101 and CD81 in collected fractions obtained after SEC separation was evaluated by Western blot, in duplicate. The detection of the sEVs protein markers TSG101 and CD81 in the samples obtained by the three isolation procedures was performed by Western blot assay. sEV aliquots were homogenized 1:1 in RIPA buffer (PBS with 1% Nonidet P-40 (#56741, Sigma-Aldrich^®^, St Louis, MO, USA), 0.5% sodium deoxycholate (#D6750, Sigma-Aldrich^®^, St Louis, MO, USA), 0.1% sodium dodecyl sulfate (Bio-Rad, #1610185, California, USA), and protease inhibitor cocktail (Roche, #04693116001, Basel, Switzerland). The homogenates were centrifuged at 10,000 × g and 4 °C for 10 min, and the supernatants were transferred to different tubes. The amount of soluble protein was quantified as described previsouly. Proteins were resuspended in reducing sodium dodecyl sulfate loading buffer and heated at 95 °C for 5 min. Next, 20 µg of sEV proteins were run on a 10% ­polyacrylamide gel under reducing conditions and transferred to a polyvinylidene fluoride membrane (Immobilon-P^®^, #IPVH00005, Merck, Darmstadt, Germany). sEVs isolated from commercial goat milk as previously described (González et al. 2023) were run in parallel as a positive control. Membranes were blocked with 3% bovine serum albumin, then incubated with an anti-TSG101 antibody (TSG101 Antibody, Rabbit, 1:1000, AB_2548734, #PA5-31260, Invitrogen, ThermoFisher Scientific, Waltham, MA, United States) or a monoclonal anti-CD81 antibody (M38, Mouse, 1:1000, AB_2532984, #10630D, Invitrogen, ThermoFisher Scientific, Waltham, MA, United States), both diluted in blocking buffer, and finally incubated with the corresponding polyclonal secondary antibodies (Immunoglobulins horseradish peroxidase–conjugated, Goat Anti-Rabbit, 1:1000, AB_2687483, #P0448, Agilent Dako, Santa Clara, CA, United States). Direct digital images were acquired with an ImageQuant LAS 4000 mini (GE Healthcare, Chicago, IL, United States).

### Cell culture

MDA-MB-231 (Human Adenocarcinoma, #92020424, European Collection of Authenticated Cell Cultures (ECACC), Porton Down, UK, CVCL_0062) and MCF-7 cell lines (Human Adenocarcinoma, HTB-22^TM^, American Type Culture Collection (ATCC), Middlesex, UK, CVCL_0031) were cultured in Dulbecco’s Modified Eagle’s Medium (DMEM, #11965092, Gibco^®^ by Life Technologies^™^, UK) supplemented with 10% Heat Inactivated Fetal Bovine Serum (FBS, #A5670701, Gibco^®^ by Life Technologies^™^, UK) and 1%(v/v) Penicillin-Streptomycin (#15140148, Gibco^®^ by Life Technologies^™^, UK), at 37 °C, 5% CO_2_. Supplemented DMEM was supplied every two to three days. Cells reaching 80% to 90% of confluence were chemically detached using 0.25% (w/v) trypsin-EDTA (#25200056, Gibco^®^ by Life Technologies^™^, UK) and seeded at the desired cell density. Counting was performed in a Neubauer chamber, using the trypan blue (#T8154, Sigma-Aldrich^®^, St Louis, MO, USA) exclusion assay.

### Cell viability assessment

Cells (MDA-MB-231, MCF-7) were seeded in 96-well plates (flat bottom, TC-treated, #734-2327, VWR Avantor^®^, Milan, Italy) (2x10^4^ cells/well) and incubated for 24h, at 37 °C, 5% CO_2_. The culture medium was replaced with different concentrations of sEVs, sEVs-SCy5, sEVs-SCy5@20-60kDa, sEVs-SCy5@250kDa and sEVs-SCy5@1000–1600kDa (at 0.63, 1.25, 2.5, 5, 10 and 20 µg/mL in supplemented DMEM). Upon 24h of incubation, the medium was removed and replaced by fresh culture medium containing 10% (v/v) of resazurin (prepared from resazurin sodium salt (#R7017, Sigma-Aldrich^®^, St Louis, MO, USA)). After 2h of incubation in the dark (37 °C, 5% CO_2_), the fluorescence of resorufin (λ_ex_ = 560 nm; λ_em_ = 590 nm) was measured in a plate reader (Biotek Instruments, Winooski, VT, USA). A negative control was included using fresh DMEM (untreated cells).

### Cellular uptake by flow cytometry

The cellular uptake of sEVs-SCy5, sEVs-SCy5@20–60kDa, sEVs-SCy5@250kDa and sEVs-SCy5@1000–1600kDa was evaluated and quantified in the same cell lines (MDA-MB-231 and MCF-7) using flow cytometry at 640 nm. Cells were seeded in 24-well plates (CytoOne 24-Well Plate, TC-treated clear, #CC7682–7524, Starlab Schweiz AG, Switzerland) (2 × 10^5^ cells per well) and incubated for 24h at 37 °C, 5% CO_2_. 200 μL of the suspensions (10 μg/mL of sEVs protein) were then added to the cells at different time points (30 min, 2h and 24h).

To screen the different cellular pathways involved in the uptake of sEVs and sEVs@1000–1600kDa, cells were also seeded in 24-well plates (2 × 10^5^ cells per well) and incubated for 24h at 37 °C, 5% CO_2_. Cells were then pretreated with different pathway inhibitors (chlorpromazine (10 μg/mL, #C8138, Sigma-Aldrich^®^, St Louis, MO, USA), hyaluronic acid (1000–1600kDa) or incubation at 4 °C). After 30 min of pretreatment, sEVs-SCy5 and sEVs-SCy5@1000–1600kDa (10 μg/mL, 200 μL) were added and left in contact with cells during 1h, at 37 °C, 5% CO_2_.

In both assays, after the defined incubation time, cells were then washed with PBS and detached using trypsin-EDTA 0.25% (w/v) and resuspended in PBS. A staining with propidium iodide (0.01 mg/mL, #P4170, Sigma-Aldrich^®^, St Louis, MO, USA) was performed to exclude dead cells. Cellular uptake was measured in a BD Accuri C6 flow cytometer at 640 nm (BD Biosciences, Erembodegem, Belgium) with at least 10,000 events acquired per sample. Data analysis was performed using the BD Accuri^™^ C6 software (BD Biosciences, Belgium).

### Confocal laser scanning microscopy

Internalization of the vesicles in MCF-7 and MDA-MB-231 cell lines was visualized using Confocal Laser Scanning Microscopy (CLSM). Cells were seeded at a density of 1 × 10^4^ cells per well in 8 well μ-slides (#80826, Ibitreat, Ibidi GmbH, Munich, Germany) and incubated at 37 °C, 5% CO_2_ overnight. Cells were then incubated with 200 μL sEVs, sEVs@20-60kDa, sEVs@250kDa and sEVs@1000–1600kDa, at 10 μg/mL of sEVs protein, for 30 min, 2h or 24h. After the respective incubation time, cells were rinsed three times with PBS, followed by a fixation step using a formalin solution (neutral buffered 10%, #HT501128, Sigma-Aldrich^®^, St Louis, MO, USA) 10 min at room temperature. For the detection of CD44, fixed cells were blocked using PBS/EDTA/BSA (2 mM EDTA disodium salt dihydrate (#E4884, Sigma-Aldrich^®^, St Louis, MO, USA) and 0.5% BSA (#A2153, Sigma-Aldrich^®^, St Louis, MO, USA)) and then incubated with CD44 antibody conjugated with FITC (CD44 Antibody, anti-human, FITC, Monoclonal Mouse IgG1, 1:50, #130-113-334, Miltenyi Biotec, Bergisch Gladbach, Germany) for 10 min, at 4 °C in the dark. Cells’ nuclei were then stained with Hoechst 33,342^®^ (8 µM in PBS, Hoechst 33,342^®^ Trihydrochloride Trihydrate, #H1399, Invitrogen, Thermo Fisher Scientific, MA, USA), performing an incubation of 10 min at room temperature. All the previously described staining steps were followed by washing procedures with PBS.

Images were acquired using a Leica Stellaris 8 confocal microscope (LeicaMicrosystems, Wetzlar, Germany) equipped with the Leica Application Suite X package (LAS X), at the Imaging by confocal and fluorescence lifetime laboratory, CEMUP, Portugal. All images were acquired using a λex of 350 nm (Hoechst 33,342^®^), 673 nm of SCy5 and 495 nm of FITC. The resolution of the images was 1024 × 1024, and the 63X/1.4 oil immersion objective was employed. The laser and acquisition parameters were maintained for conditions intended to be compared.

To automate the analysis of the CLSM images a Python script was created. Users are prompted to manually annotate the cell count for each image. Subsequently, based on whether the images relate to sEVs uptake or immunofluorescence, the script considers the red or green channels, respectively. The total pixel intensity of the red/green channel is then divided by the number of cells in the respective image, providing a more standardized metric for the fluorophore intensity per cell. This process was executed on a minimum of 8 images for each condition, with at least 5 cells per image.

### Statistical analysis

Prism software v9.5.1 (GraphPad Software Inc., CA, USA) was used for statistical analysis. The data underwent analysis through one-way analysis of variance (ANOVA) or two-way ANOVA, and Tuckey’s multiple comparison. Statistical significance was set as follows: *(*p* ≤ 0.05), **(*p* ≤ 0.01), ***(*p* ≤ 0.001), ****(*p* ≤ 0.0001). Results are presented as mean ± standard deviation (SD).

## Results

### Isolation and characterization of milk extracellular vesicles

The employed isolation method yielded a total protein content of 3.98 ± 0.28 µg/mL of bovine milk and a total number of sEVs per µg of protein of 2.5 x 10^9^ ± 1.1 x 10^8^, which corresponds to 9.9 x 10^9^ ± 2.7 x 10^8^ sEVs/mL milk. Higher yields (approximately 10^11^ sEVs/ml of starting material) have been reported for alternative methods based, for example, on the removal of casein by acidification or chelation (Somiya et al. 2018; Vaswani et al. 2019). However, it is noteworthy that these methods also reported higher protein contents, exceeding 13 µg/ml of milk (Somiya et al. 2018). The relatively lower protein yield in our method may be attributed to a more effective elimination of non-sEVs proteins. Additionally, sEVs isolates were estimated to have approximately 10^9^ particles per µg of protein, a value consistent with the values obtained with our method (Sverdlov 2012).

**Figure 1.**
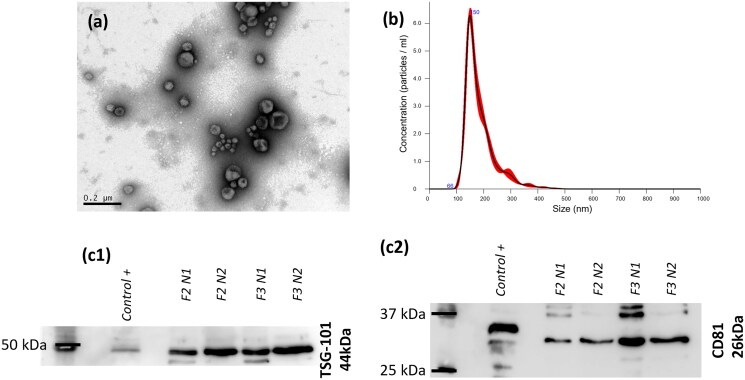
Physicochemical characterization of the isolated sEVs: (a) TEM image for morphological evaluation (100 000x); (b) Nanoparticle tracking analysis size distribution of a representative sample; detection of (C1) TSG101 and (C2) CD81 in the isolates (F2 and F3, in duplicate), by Western blot (cropped gels), compared to a positive control of sEVs obtained from goat milk.

Regarding the size distribution of the isolated sEVs, NTA showed a relatively monodisperse population with a mean size of 186 ± 4 nm and a mode size of 148 ± 5 nm (Table 1). A representative size distribution obtained with NTA is shown in Figure 1(b). Although we obtained a slightly larger mean size than reported in the literature for sEVs, this is most likely due to some aggregation, as can be seen in the TEM images (Figure 1a). The observed ‘cup-shaped’ morphology of the vesicles, indicating some deformation from a spherical shape, is characteristic of these structures when examined using electron microscopy techniques like TEM (Sedykh et al. 2020). This is due to the dehydration and vacuum conditions involved, which can distort their native round form. The TEM image also suggests that the isolated sEVs exhibit high purity, as no co-isolated non-vesicular extracellular particles are detectable. The results of the Western blot are shown in Figure 1 (C1) and (c2) (complete gels are presented in Figure S1 in Supplementary Material). The samples tested consisted of fractions 2 and 3 collected the purification step, in two independent isolation procedures (N1 and N2). For TSG101, all bands are at 44 kDa, as in the positive control. For CD81, all tested samples show a band at 26 kDa corresponding to the CD81 protein. However, the band in the positive control appears slightly shifted to a higher MW, which may be due to non-specific binding, potentially caused by the antibody not being validated for bovine samples. This deviation could also be due to post-translational modifications. Despite this shift, we are confident that the band corresponds to CD81, as it aligns with the expected MW of the protein. It can be confirmed that all samples contain TSG101 and CD81 in their composition, which are known sEVs biomarkers. The sEVs have therefore been successfully isolated.

**Table 1.** Size characterization of sEVs, sEVs-SCy5, sEVs-SCy5@20-60kDa, sEVs-SCy5@250kDa, sEVs-SCy5@1000–1600kDa.

	sEVs	sEVs-SCy5	sEVs-SCy5@20–60kDa	sEVs-SCy5@250kDa	sEVs-SCy5@1000–1600kDa
Mean Size (nm)	186 ± 4	173 ± 3	178 ± 3	183 ± 2	185 ± 2
Mode Size (nm)	148 ± 4	152 ± 9	154 ± 5	180 ± 5	163 ± 5

### Fluorescence labeling and functionalization

For the fluorescent detection of sEVs, a commercially available fluorophore (sulfo-cyanine-5-NHS ester) was first chemically linked to the ionized amine groups of the surface proteins *via* a covalent bond (Santos‐Coquillat et al. 2022). This simple method was also used for the functionalization with HA, but in this case, activated HA (HA-NHS ester) was used as the binding molecule.

**Figure 2.**
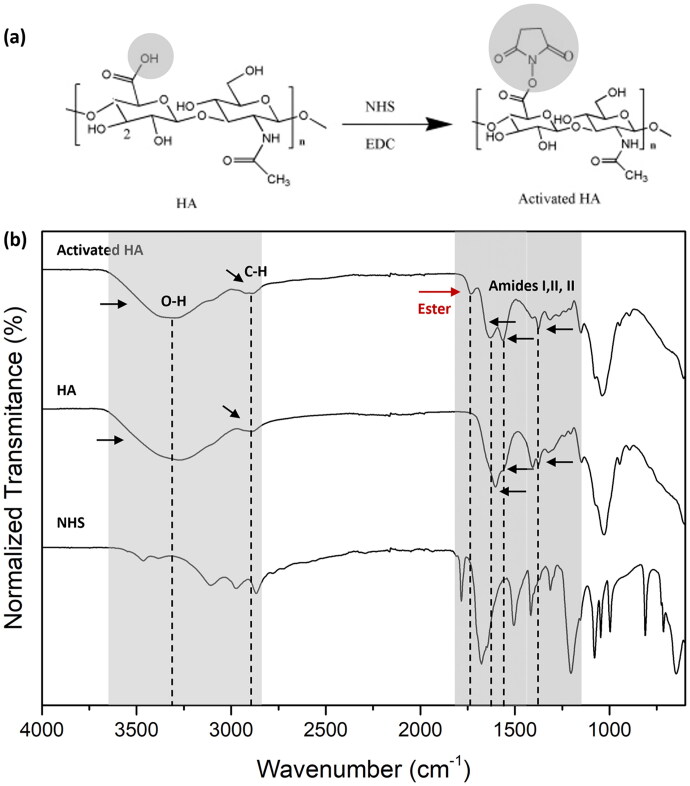
(a) Schematic representation of the reaction of activation of HA carboxylic acid groups using carbodiimide chemistry. (b) FTIR spectra of activated HA (HA-NHS-ester), HA and NHS. The characteristic absorption peaks are indicated with arrows; the red arrow highlights the new peak corresponding to the formation of an ester bond.

Activated HA was synthesized and Figure 2(b) shows the FTIR spectrum of the obtained product as well as the spectra of HA and NHS. Since all HA MW yielded similar spectra, only one representative example of HA and activated HA is shown. The typical peaks of HA appear in the recorded spectrum: a broad band around 3400 cm^−1^ attributable to hydrogen-bonded O-H and N-H stretching vibrations; at about 2900 cm^−1^ a peak corresponding to C-H stretching vibrations; at about 1650, 1560 and 1320 cm^−1^ characteristic peaks of amides I, II and III, respectively; peak around 1728 cm^−1^ attributable to the carboxyl groups (Zhang et al. 2019). Similar peaks were identified in the spectra of the activated HA. However, the appearance of a new peak at 1800 cm^−1^ corresponding to the ester group as well as the intense reduction of the OH band, indicates the successful synthesis of HA NHS ester. Activated HA with different MW was used for the functionalization of sEVs previously labeled with SCy5.

After surface modification of the sEVs mean and mode sizes, obtained by NTA, did not change drastically (Table 1). Functionalization with 250 kDa HA and 1000–1600kDa HA slightly increased sEVs mode size, which means that the distribution of the larger HA molecules on sEVs surface must be on a thin layer, possibly with several binding points between the same HA molecules with different groups of the sEVs surface. Also, the morphology of the sEVs was maintained after the labelling and functionalization process, as observed by TEM (Figure S2).

### Cytocompatibility evaluation on cancer cell lines

**Figure 3.**
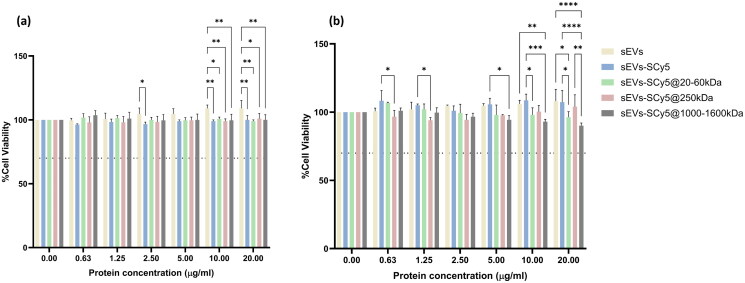
*In Vitro* metabolic activity evaluation with (a) MCF-7 and (b) MDA-MB-231, determined by the resazurin assay. Both cell lines were treated with different concentrations of sEVs, sEVs-SCy5, sEVs-SCy5@20-60kDa, sEVs-SCy5@250kDa and sEVs-SCy5@1000–1600kDa, for 24h. Reference data is represented by the results with untreated cells (0 µg/ml of protein). Statistical analysis was performed using the two-way ANOVA and Tukey’s multiple comparisons test (*p ≤ 0.05, **p ≤ 0.01, ***p ≤ 0.001, ****p ≤ 0.0001).

Prior to all further *in vitro* tests, the cytocompatibility of the isolated and engineered sEVs was tested using the resazurin assay (Figure 3). The cell lines (MDA-MB-231 and MCF-7) were incubated for 24h with different concentrations of sEVs, sEVs-SCy5, sEVs-SCy5@20-60kDa, sEVs-SCy5@250 kDa and sEVs-SCy5@1000–1600kDa. Regardless of the dosage, labeling and functionalization, it was found that cell viability was maintained at approximately 100%. Interestingly, the naked sEVs and sEVs-SCy5 at higher concentrations led to an increase in metabolic activity compared to the same concentration of the other sEVs formulations. A similar result was previously observed with other sEVs isolated from milk (Cao et al. 2019; Santos‐Coquillat et al. 2022). This could be attributed to the ability of these vesicles to regulate and assist in the recovery from various types of cellular damage, promoting an increase in metabolic activity and even cell growth (Arntz et al. 2015; Wang et al. 2021; Mecocci et al. 2022). The same result does not occur with functionalized sEVs. They appear to preserve cellular metabolic activity at levels comparable to the control, effectively counteracting the previously observed effect. This offers an advantage over unmodified sEVs, as the carrier itself should not enhance cancer cell metabolism in nanomedicine applications.

Overall, none of the formulations tested induced cytotoxicity, so further studies could be carried out under these conditions.

### Assessment of CD44 expression levels on breast cancer cell lines

As we intend to apply our strategy to target breast cancer *via* CD44, we focused our attention on the detection of this biomarker on the cell membrane of two different breast cancer (BC) cell lines: MCF-7, representing the hormone receptor-positive (HR+) breast cancer subtype, and MBA-MB-231, corresponding to TNBC. The expression of CD44 was analyzed by immunofluorescence on both cell lines.

**Figure 4.**
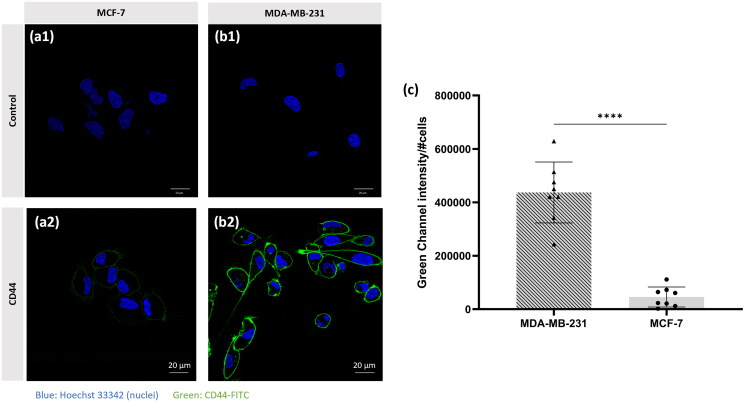
Immunofluorescence of CD44 protein (FITC - green) on (a2) MCF-7 and (b2) MDA-MB-231 BC cell lines. CLSM control images of (a1) MCF-7, (b1) MDA-MB-231 cells. Cell nuclei were stained with Hoechst 33,342 dye (blue). (c) Quantification of the green channel intensity per number of cells. The analysis was carried out in 8 images of each cell line, with a minimum of 5 cells per image. Statistical analysis was performed using unpaired t-test (****p ≤ 0.0001).

This analysis revealed that the expression of CD44 differs greatly between the cell lines examined (Figure 4). The TNBC cell line (MDA-MB-231) showed higher CD44 expression, while the MCF-7 cell line showed almost no expression. In terms of fluorescence intensity, an 8-fold higher signal is given by the MDA-MB-231 cells in comparison with the MCF-7 cells. This result is consistent with the results reported in the literature for these cell lines (Sheridan et al. 2006; Qhattal and Liu 2011). Furthermore, it supports the hypothesis that the expression of CD44 correlated positively with invasiveness and metastatic capacity (Sheridan et al. 2006).

Based on this information, we were able to classify the MCF-7 cell line as a cell line with low CD44 expression (CD44-) and the MDA-MB-231 as a cell line with high CD44 expression (CD44+), so that we can discuss the results of internalization of sEVs accordingly. This is a very important aspect as we intend our nanosystems to undergo selective internalization by membrane CD44 receptors following HA binding.

### Binding and internalization evaluation through confocal microscopy

To evaluate the cellular uptake of the non-functionalized and functionalized sEVs, the cell lines MDA-MB-231 and MCF-7 were used as CD44+ and CD44− cells, respectively. For these studies, cells were incubated with 200 µL of sEVs (functionalized and non-functionalized) in a concentration of 10 µg/mL, for 30 min, 2h and 24h.

**Figure 5.**
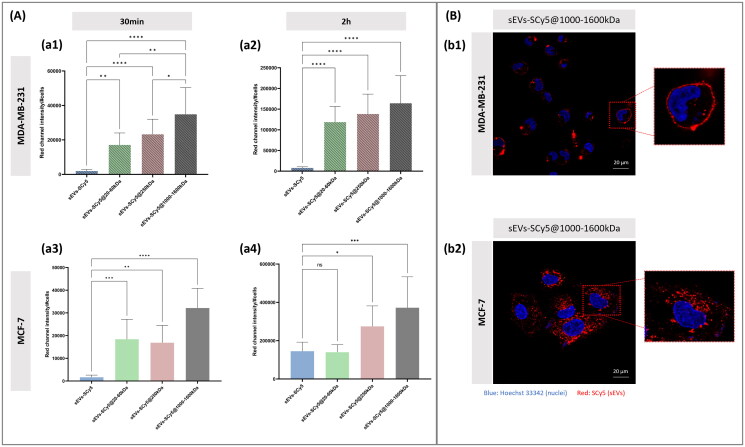
(A) Evaluation of sEVs (functionalized and non-functionalized) uptake by MDA-MB-231 cell line after (a1) 30 min and (a2) 2h; and MCF-7 cell line after (a3) 30 min and (a4) 2h. Quantification of fluorescent sEVs on CLSM images (red channel intensity per number of cells). The analysis was carried out in 8 images of each cell line, with a minimum of 5 cells per image. Statistical analysis was performed using the two-way ANOVA and Tukey’s multiple comparisons test (*p ≤ 0.05, **p ≤ 0.01, ***p ≤ 0.001, ****p ≤ 0.0001). (B) CLSM images of MDA-MB-231 (b1) and MCF-7 (b2) cell lines after 2h of incubation with sEVs-SCy5@1000–1600kDa. Cell nuclei were stained with Hoechst 33342 dye (blue) and functionalized and non-functionalized sEVs were labeled with SCy5 (red). Zoomed-in cells are presented on the right side of each image to facilitate visualization of the sEVs distribution on cells.

Firstly, we sought to assess the impact of increasing HA MW for shorter incubation periods. For this purpose, sEVs functionalized with HA with different MW (20–60kDa, 250 kDa, 1000–1600kDa) were used. Quantitative analysis of the CLSM images (Figure 5A) revealed that non-functionalized sEVs exhibited significantly lower uptake by both cell lines regardless of the incubation time. For instance, for the MDA-MB-231 cell line, the fluorescence intensity per cell for the non-functionalized sEVs remained around 4500 for 30 min of incubation and 40,000 for 2h of incubation, which is one order of magnitude lower than the functionalized sEVs, at the same time point. However, in the case of MCF-7 cells, after a 2h-incubation, the difference between non-functionalized and functionalized sEVs decreased, becoming nonsignificant for sEVs-SCy5 and sEVs-SCy5@20–60kDa. Conversely, in the case of the MDA-MB-231 cell line, all HA MW resulted in a significantly increased uptake of sEVs, with a slight tendency toward higher values for higher HA MW (with a mean fluorescence intensity per cell of approximately 250,000 compared to the 230,000 for sEVs@250kDa and 120,000 for sEVs@20–60kDa). Despite the lower CD44 expression levels, MCF-7 appears to internalize higher quantities of functionalized sEVs when compared to non-functionalized ones. The highest fluorescence was also observed for sEVs-SCy5@1000–1600 kDa, but in the case of this CD44- cell line, showing a modest 2-fold increase over the non-functionalized counterpart.

It is important to highlight that sEVs with different labeling efficiencies were used for each cell line, for 30 min and 2 h incubations. Therefore, even with identical acquisition parameters, direct comparisons of fluorescence values were only conducted within the same cell line and between different timepoints, and not between cell lines, under these conditions.

As shown in Figure 5B, it can be observed that after a 2h-incubation, sEVs-SCy5@1000–1600kDa in MCF-7 cells exhibit a uniform distribution throughout the cytoplasm. In contrast, the distribution of sEVs in MDA-MB-231 cells is predominantly along the cell surface, which may indicate that the sEVs-SCy5@1000–1600kDa are mainly bound to the surface at this time point.

This suggests that a membrane receptor, such as CD44, plays a key role in the binding of sEVs-SCy5@1000–1600 kDa to CD44+ cells. In contrast, the uptake observed in CD44− cells likely occurs through alternative, receptor-independent pathways.

**Figure 6.**
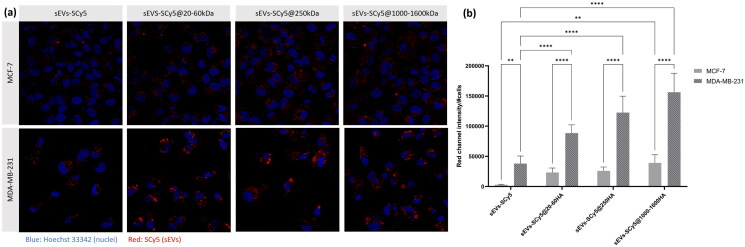
Evaluation of sEVs (functionalized and non-functionalized) uptake by MCF-7 and MDA-MB-231 BC cell lines, after 24h of incubation. (a) CLSM images of BC cell lines after 24h of incubation with 10 μg/ml of sEV-SCy5, sEVs-SCy5@20-60kDa, sEVs-SCy5@250kDa and sEVs-SCy5@1000–1600kDa. Cell nuclei were stained with Hoechst 33,342 dye (blue) and functionalized and non-functionalized sEVs were labeled with SCy5 (red). (b) Quantification of fluorescent sEVs on CLSM images (red channel intensity per number of cells). The analysis was carried out in 8 images of each cell line, with a minimum of 5 cells per image. Statistical analysis was performed using the two-way ANOVA and Tukey’s multiple comparisons test (*p ≤ 0.05, **p ≤ 0.01, ***p ≤ 0.001, ****p ≤ 0.0001).

In order to effectively compare the uptake between the cell lines, an additional incubation time (24h), was investigated using the same batch of labelled sEVs. The corresponding results are shown in Figure 6. Again, the internalization of functionalized sEVs by MDA-MB-231 cells was significantly increased (around 4-fold higher fluorescence intensity) compared to MCF-7 cells.

In the case of MDA-MB-231 cells, the trend of increased uptake for higher HA MW, which was already evident at previous time points, is confirmed by quantitative analysis of CLSM images after 24 h of incubation. The difference is further supported by fluorescence quantification, with MCF-7 cells showing fluorescence values around 40,000, compared to approximately 150,000 for MDA-MB-231 cells when incubated with sEVs@1000–1600 kDa. Although the uptake of functionalized sEVs is enhanced in MCF-7 (CD44-) cells compared to non-functionalized counterparts, the increase in fluorescence observed in MDA-MB-231 cells is markedly more pronounced, highlighting the impact of CD44-mediated interactions in promoting more efficient internalization of functionalized sEVs. Consequently, the functionalization of sEVs demonstrates strong potential for selective targeting of CD44+ cells, with high HA MW showing the most pronounced increase in uptake selectivity.

Notably, in CD44+ cells, sEVs-SCy5@1000–1600 kDa were observed within the cytoplasm after 24 h of incubation (Figure 6a), indicating that these vesicles are internalized following their membrane binding (Figure 5b). This supports the hypothesis that a membrane receptor like CD44 mediates binding and is functionally active, enabling vesicle internalization.

### Internalization assessed via flow cytometry

**Figure 7.**
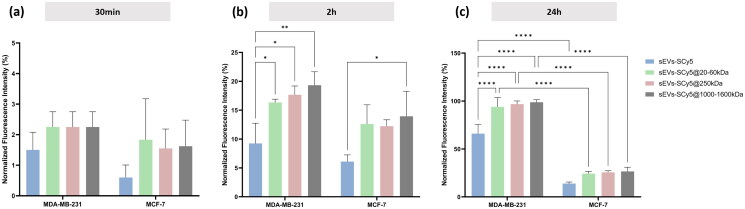
Evaluation of sEVs-SCy5, sEVs-SCy5@20-60kDa, sEVs-SCy5@250kDa, sEVs-SCy5@1000–1600kDa internalization by MDA-MB-231 and MCF-7 cell lines, after trypsinization. Flow cytometry results corresponding to incubation times of (a) 30 min, (b) 2h and (c) 24h, of each type of sEVs (10 µg/ml). Statistical analysis was performed using the two-way ANOVA and tukey’s multiple comparisons test (*p ≤ 0.05, **p ≤ 0.01, ***p ≤ 0.001, ****p ≤ 0.0001).

The CLSM results shown above include both cell surface binding and internalization of sEVs. To evaluate internalization only, BC cells were incubated with both non-functionalized and functionalized sEVs (for 30 min, 2h, and 24h), and fluorescence was quantified by flow cytometry after trypsin treatment. Trypsin has been reported to degrade CD44 and consequently release CD44-bound material from the cell surface (Rios de la Rosa et al. 2019). Thus, monitoring fluorescence under these conditions allows selective quantification of vesicles inside the cell. The results obtained are shown in Figure 7.

Comparable internalization kinetics can be observed in all conditions during the first 30 min, a trend that begins to change after 2h of incubation. At this time point, the internalization rates between the MDA-MB-231 and MCF-7 cell lines are relatively similar. However, there are differences in internalization values between non-functionalized sEVs and their functionalized counterparts, with 2-fold higher fluorescence measured for higher HA MW when compared to the unmodified sEVs. After 24 h of incubation, internalization by MDA-MB-231 cells is markedly higher than in MCF-7 cells, with normalized fluorescence intensities reaching approximately 100% in MDA-MB-231 compared to only 20% in MCF-7. These data are consistent with the CLSM analysis for a 24h-incubation (Figure 6b). However, while the CLSM analysis for the MDA-MB-231 cell line showed significant differences between all sEVs formulations, in this case, all HA MW gave similar fluorescence intensities. This suggests that there are different binding and internalization dynamics in this cell line depending on which HA MW is used for functionalization.

### Cellular uptake pathways assessment through flow cytometry

**Figure 8.**
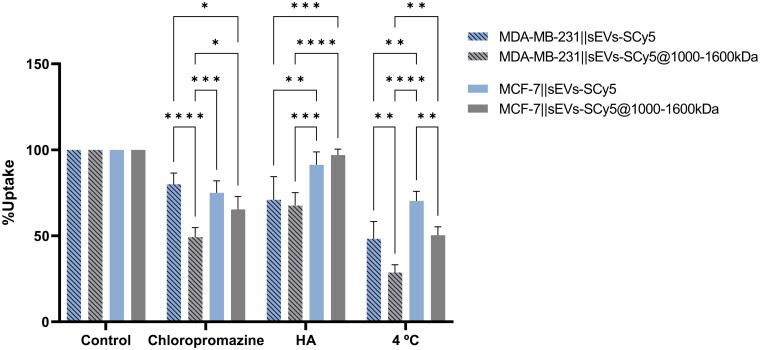
Assessment of cellular internalization pathways of sEVs-SCy5 and sEVs-SCy5@1000–1600kDa on MDA-MB-231 and MCF-7 cell lines, by flow cytometry. Cells were pre-incubated with the respective pathway inhibitor (chlorpromazine and HA 1000–1600kDa) or incubated at 4 °C for 30 min before incubation with sEVs-SCy5/sEVs-SCy5@1000–1600kDa for 1 h. Fluorescence values were normalized to those of cells (of the same cell line) without any inhibition. Statistical analysis was performed using the two-way ANOVA and Tukey’s multiple comparisons test (*p ≤ 0.05, **p ≤ 0.01, ***p ≤ 0.001, ****p ≤ 0.0001).

Binding and internalization studies revealed significant differences between BC cell lines, suggesting the potential involvement of CD44 in the selective uptake of functionalized sEVs. To investigate the internalization pathways of both functionalized and non-functionalized vesicles, sEVs-SCy5 and sEVs-SCy5@1000–1600HA were selected for further studies. MDA-MB-231 and MCF-7 cells were exposed to different conditions aimed at inhibiting specific pathways (chlorpromazine, HA 1000–1600kDa, or incubation at 4 °C) and subsequently incubated with sEVs-SCy5 and sEVs-SCy5@1000–1600HA in the presence of each inhibitor. The fluorescence was once again assessed by flow cytometry after trypsinization, ensuring that the results exclusively reflect internalization (Figure 8). All the inhibition strategies used were aimed at evaluating the involvement of the CD44 receptor in the uptake process.

Since CD44 is an endocytic receptor (Rios de la Rosa et al. 2019), chlorpromazine was used to inhibit clathrin-mediated endocytosis, which is traditionally associated with receptor-mediated endocytosis (Rennick et al. 2021). A decrease in uptake between non-functionalized and functionalized sEVs was observed in both cell lines. Nevertheless, a 30% decrease was observed in MDA-MB-231 cells, compared to a modest 10% reduction in MCF-7 cells. This more pronounced decrease in MDA-MB-231 suggests a greater involvement of endocytic mechanisms in the uptake of sEVs-SCy5@1000–1600HA by this cell line. A similar behavior can be observed when the experiment is performed at 4 °C. However, in this case, the uptake of sEVs-SCy5 also seems to be affected, albeit to a lesser extent. This suggests that active energy-dependent processes may be involved in the uptake of both types of vesicles. Some surface proteins on sEVs have been shown to enhance non-selective receptor-mediated endocytosis, contributing to their cellular uptake (Gonda et al. 2019). Additionally, reduced temperatures are known to increase membrane rigidity, which may hinder membrane fusion, a process also proposed as a key mechanism for sEV internalization (Gonda et al. 2019).

Pre-incubation with free HA (MW 1000–1600 kDa) aimed to saturate the CD44 receptors yielded interesting results. Contrary to our expectations, the difference in uptake between non-functionalized and functionalized sEVs was not significant within the same cell line. However, a higher decrease in uptake was observed in MDA-MB-231 compared to MCF-7, for both types of vesicles. This effect may be attributed to the differing levels of CD44 expression between the cell lines. In cells with higher CD44 expression, pre-incubation with HA can saturate a substantial portion of the available receptors, thereby hindering the binding and uptake of HA-functionalized sEVs. Moreover, the presence of excess HA (a large molecule) on the cell surface may physically interfere with other internalization routes, such as membrane fusion. This steric hindrance could also reduce the uptake of non-functionalized sEVs. In contrast, cells lacking CD44 are less affected by HA pre-incubation, which may explain their higher sEV uptake under these conditions.

In summary, CD44 appears to play a role in the internalization of sEVs-SCy5@1000–1600kDa, involving endocytic, energy-dependent mechanisms. In addition, potential uptake mechanisms for non-functionalized sEVs were uncovered, involving membrane fusion-dependent pathways or even non-selective receptor-mediated endocytosis.

## Discussion

Cancer nanomedicine has witnessed significant advancements in recent decades, with sEVs emerging as promising vehicles for drug delivery. Their natural origin, inherent biocompatibility, and enhanced stability, in contrast to synthetic liposomes, contribute to their appeal. Moreover, milk has demonstrated potential as a scalable and abundant source of sEVs, with millions of tons produced annually, predominantly by cows (Soares et al. 2023). Thus, particularly bovine milk, has emerged as a cost-effective and abundant source of sEVs, offering significantly higher yields than human cell-derived sources. In the context of nanomedicine, milk-derived sEVs (from non-human sources) offer notable advantages, particularly their lack of coagulation factors, which is beneficial for intravenous administration by reducing the risk of thrombotic complications (Hu et al. 2020). Moreover, these vesicles have demonstrated intrinsic therapeutic properties, including positive effects on intestinal health, skin and hair regeneration, and the treatment of bone-related disorders (Ong et al. 2021).

However, the lack of standardization in sEVs isolation methods poses a significant hurdle in their application. A standardized approach is essential not only for ensuring robust and reproducible research outcomes but also for advancing the translational potential of sEVs in nanomedicine. In this sense, our work takes a step in this direction by providing comprehensive characterization, including size, morphology, and protein markers, along with purity and yield indicators, aiming to enhance comparability with other methods. Despite yielding a lower particle count per milliliter of starting material compared to alternative methods, our approach holds promise in efficiently eliminating non-sEVs proteins, and potentially higher purity.

Recent studies have highlighted the promising role of milk-derived vesicles in cancer therapy. Pioneering work by Munagala and colleagues showcased the versatility of sEVs to carry diverse anticancer molecules, and enhance the efficacy of antitumor drugs, while resisting gastrointestinal conditions (Munagala et al. 2016, 2017). In particular, these vesicles demonstrated potential for tumor targeting when decorated with folic acid (Munagala et al. 2016). Other researchers have explored similar strategies using folic acid (Aqil et al. 2019) and alternative targeting molecules, including HA (Li et al. 2020, 2022; Cui et al. 2022), iRGD peptide (Pullan et al. 2022), and GE11 peptide (Go et al. 2022). Nevertheless, functionalization approaches have relied on the passive integration of lipid-modified molecules, like DSPE-PEG, into the sEVs membrane. These methods require the use of organic solvents, which may compromise the stability of sEVs. Also, it may introduce structural changes to the sEVs by incorporating new lipid molecules on the bilayer. Additionally, the functionalization efficiency tends to be low, which demands long incubation times. In contrast, our approach involves the covalent binding of HA to sEVs, offering advantages over passive integration. It is based on the chemical reaction between the available ester groups of the activated HA and the amine groups present in surface proteins of the nanovesicles, under slightly basic pH. This straightforward methodology has been previously described for labelling and surface modification with radioisotopes (González et al. 2020, 2021; Santos‐ Coquillatet al. 2022) and in this work, we apply it for the optical labeling with SCy5 and functionalization with HA. This covalent linkage ensures a robust and stable bond between the sEVs and the ligand molecules, minimizing the risk of detachment. Moreover, for the functionalization, this method eliminates the need for organic solvents and prevents structural changes to the sEVs, providing a more controlled and efficient functionalization process. From our results, any significant size changes were found after labelling and functionalization of sEVs, showing that this approach may hold great promise in the scope of sEVs surface modification.

A topic that remains relatively under-discussed is the influence of HA MW on the targeting capabilities of functionalized sEVs. Existing studies either focus only on one HA MW (typically 30 kDa) (Li et al. 2020, 2022) or do not provide information on the HA MW that was used (Cui et al. 2022). Consequently, the current understanding of how HA MWs affect the binding and internalization dynamics of sEVs remains limited. In fact, it is known that structural alterations in HA molecules can influence their binding and internalization capacities towards CD44 (Bhattacharya et al. 2017). Thus, in this work, we sought to address this knowledge gap by exploring three distinct HA MW to discern and compare their effects on cellular uptake dynamics. Overall, our results show that higher MW led to higher levels of interaction between functionalized sEVs and CD44+ cells. These results are consistent with recent studies reporting that the higher the MW value of the HA molecules, the higher the affinity for CD44, which is due to the existence of multivalent binding interactions (Guo et al. 2022). Further investigation of the binding affinity of HA to CD44 after chemical activation and conjugation to sEVs, along with the impact of HA MW on targeting efficiency, using molecular docking methods, could provide valuable insights and facilitate comparison with experimental results. It is important to note, however, that this increased interaction does not seem to translate directly into internalization. This appears to be more pronounced for higher HA MW, as the discrepancy between binding and internalization outcomes is more significant in this case. A possible explanation for this observation lies in the stronger binding affinity of higher MW HA to CD44. While this may enhance receptor-mediated uptake by promoting CD44 clustering (Zhong et al. 2019), it could simultaneously hamper alternative internalization routes by restricting direct sEV-membrane interactions. Despite this potential trade-off, the internalization of HA-functionalized sEVs by MDA-MB-231 cells remains markedly higher than that of non-functionalized vesicles and significantly exceeds uptake levels observed in MCF-7 cells, thereby supporting our hypothesis. It is noteworthy that this study did not investigate the role of CD44 isoforms. Rather, it focused on assessing the impact of the overall overexpression of CD44 in TNBC cells, for targeting purposes. Some studies have been exploring the interactions between HA molecules of varying MW and different CD44 isoforms (Spadea et al. 2019; Kim and Owen 2020). Thus, as a future approach, it would be also interesting to shed some light on how distinct CD44 isoforms might interact with functionalized sEVs.

## Conclusions

In summary, our investigation has unveiled an alternative for cancer targeting, particularly for TNBC, that still lacks broadly effective targeted treatments. By exploiting sEVs derived from cow milk and functionalizing them with HA, we have been able to target the overexpressed CD44 cell surface receptor in TNBC cells.

This investigation introduces an innovative methodology for cow milk sEVs isolation and functionalization. The functionalization with HA of varying MW not only demonstrated enhanced selectivity for CD44+ cells but also revealed the impact of HA MW on binding and internalization dynamics. Our results indicated that higher MW HA conferred superior binding capacity, and partial internalization through CD44 endocytic mechanisms.

These insights contribute to the development of improved sEVs-based therapies, particularly for TNBC, addressing several research gaps and critical challenges in cancer nanomedicine. The use of HA-functionalized milk-derived sEVs represents a compelling strategy for targeted drug delivery. By selectively directing therapeutic agents to CD44-overexpressing cells, this approach enhances treatment specificity and efficacy, while potentially reducing off-target effects. This is especially valuable for TNBC patients, who often lack access to current targeted therapies due to their genetic profile. Beyond therapeutic applications, these engineered nanocarriers also offer diagnostic and prognostic potential. Their radiolabeling could enable early and precise tumor detection *via* nuclear imaging techniques. Detection of CD44-overexpressing breast tumors may offer valuable insights into selecting the most effective therapeutic strategies, as well as into predicting disease progression and treatment responsiveness. Overall, this platform paves the way for more effective and personalized approaches in oncology, especially for TNBC patients.

## Supplementary Material

Supplementary_material.pdf

## Data Availability

Data supporting the results reported in the article can be found in supplementary material.

## References

[CIT0001] Adriano B, Cotto NM, Chauhan N, Jaggi M, Chauhan SC, Yallapu MM. 2021. Milk exosomes: nature’s abundant nanoplatform for theranostic applications. Bioact Mater. 6(8):2479–2490. doi: 10.1016/j.bioactmat.2021.01.009. 33553829 PMC7856328

[CIT0002] Aqil F, Munagala R, Jeyabalan J, Agrawal AK, Kyakulaga A-H, Wilcher SA, Gupta RC. 2019. Milk exosomes-natural nanoparticles for siRNA delivery. Cancer Lett. 449:186–195. doi: 10.1016/j.canlet.2019.02.011. 30771430

[CIT0003] Arntz OJ, Pieters BCH, Oliveira MC, Broeren MGA, Bennink MB, de Vries M, van Lent PLEM, Koenders MI, van den Berg WB, van der Kraan PM, et al. 2015. Oral administration of bovine milk derived extracellular vesicles attenuates arthritis in two mouse models. Mol Nutr Food Res. 59(9):1701–1712. doi: 10.1002/mnfr.201500222. 26047123

[CIT0004] Bhattacharya DS, Svechkarev D, Souchek J, Hill TK, Taylor M, Natarajan A, Mohs AM. 2017. Impact of structurally modifying hyaluronic acid on CD44 interaction. J Mater Chem B. 5(41):8183–8192. doi: 10.1039/C7TB01895A. 29354263 PMC5773055

[CIT0005] Cadete A, Alonso MJ. 2016. Targeting cancer with hyaluronic acid-based nanocarriers: recent advances and translational perspectives. Nanomedicine . 11(17):2341–2357. doi: 10.2217/nnm-2016-0117. 27526874

[CIT0006] Cao L, Xu H, Wang G, Liu M, Tian D, Yuan Z. 2019. Extracellular vesicles derived from bone marrow mesenchymal stem cells attenuate dextran sodium sulfate-induced ulcerative colitis by promoting M2 macrophage polarization. Int Immunopharmacol. 72:264–274. doi: 10.1016/j.intimp.2019.04.020. 31005036

[CIT0007] Chapdelaine AG, Sun G. 2023. Challenges and opportunities in developing targeted therapies for triple negative breast cancer. Biomolecules. 13(8):1207. doi: 10.3390/biom13081207. 37627272 PMC10452226

[CIT0008] Cui W, Tie S, Guo M, Qiao F, Tan M, Su W. 2022. Engineering milk-derived exosome for enhancing cellular astaxanthin delivery. J Agric Food Chem. 70(35):10794–10806. doi: 10.1021/acs.jafc.2c03683. 36018242

[CIT0009] Fu C-P, Cai X-Y, Chen S-L, Yu H-W, Fang Y, Feng X-C, Zhang L-M, Li C-Y. 2023. Hyaluronic acid-based nanocarriers for anticancer drug delivery. Polymers . 15(10):2317. doi: 10.3390/polym15102317. 37242892 PMC10224391

[CIT0010] Go G, Park HJ, Lee JH, Yun CW, Lee SH. 2022. Inhibitory effect of oxaliplatin-loaded engineered milk extracellular vesicles on tumor progression. Anticancer Res. 42(2):857–866. doi: 10.21873/anticanres.15543. 35093883

[CIT0011] Gonda A, Kabagwira J, Senthil GN, Wall NR. 2019. Internalization of exosomes through receptor-mediated endocytosis. Mol Cancer Res. 17(2):337–347. doi: 10.1158/1541-7786.MCR-18-0891. 30487244

[CIT0012] González MI, Gallardo B, Cerón C, Aguilera-Jiménez E, Cortes-Canteli M, Peinado H, Desco M, Salinas B. 2023. Isolation of goat milk small extracellular vesicles by novel combined bio-physical methodology. Front Bioeng Biotechnol. 11:1197780. doi: 10.3389/fbioe.2023.1197780. 37829562 PMC10564981

[CIT0013] González MI, González-Arjona M, Santos-Coquillat A, Vaquero J, Vázquez-Ogando E, de Molina A, Peinado H, Desco M, Salinas B. 2021. Covalently labeled fluorescent exosomes for in vitro and in vivo applications. Biomedicines. 9(1):81. doi: 10.3390/biomedicines9010081. 33467033 PMC7829962

[CIT0014] González MI, Martín-Duque P, Desco M, Salinas B. 2020. Radioactive labeling of milk-derived exosomes with 99mTc and in vivo tracking by SPECT imaging. Nanomaterials. 10(6):1062. doi: 10.3390/nano10061062. 32486215 PMC7352469

[CIT0015] Guo Q, Yang C, Gao F. 2022. The state of CD44 activation in cancer progression and therapeutic targeting. Febs J. 289(24):7970–7986. doi: 10.1111/febs.16179. 34478583

[CIT0016] Hu Y, Hell L, Kendlbacher RA, Hajji N, Hau C, van Dam A, Berckmans RJ, Wisgrill L, Ay C, Pabinger I, et al. 2020. Human milk triggers coagulation via tissue factor-exposing extracellular vesicles. Blood Adv. 4(24):6274–6282. doi: 10.1182/bloodadvances.2020003012. 33351123 PMC7756996

[CIT0017] Jain V, Kumar H, Anod HV, Chand P, Gupta NV, Dey S, Kesharwani SS. 2020. A review of nanotechnology-based approaches for breast cancer and triple-negative breast cancer. J Controlled Release. 326:628–647. doi: 10.1016/j.jconrel.2020.07.003. 32653502

[CIT0018] Kim SJ, Owen SC. 2020. Hyaluronic acid binding to CD44S is indiscriminate of molecular weight. Biochim Biophys Acta Biomembr. 1862(9):183348. doi: 10.1016/j.bbamem.2020.183348. 32428448

[CIT0019] Li D, Gong L, Lin H, Yao S, Yin Y, Zhou Z, Shi J, Wu Z, Huang Z. 2022. Hyaluronic acid-coated bovine milk exosomes for achieving tumor-specific intracellular delivery of miRNA-204. Cells. 11(19):3065. doi: 10.3390/cells11193065. 36231028 PMC9562169

[CIT0020] Li D, Yao S, Zhou Z, Shi J, Huang Z, Wu Z. 2020. Hyaluronan decoration of milk exosomes directs tumor-specific delivery of doxorubicin. Carbohydr Res. 493:108032. doi: 10.1016/j.carres.2020.108032. 32417443

[CIT0021] Liu D, Liu T, Li R, Sy M-S. 1998. Mechanisms regulating the binding activity of CD44 to hyaluronic acid. Front Biosci. 3(4):d631–636. doi: 10.2741/A307. 9634638

[CIT0022] Liu X, Xiao C, Xiao K. 2023. Engineered extracellular vesicles-like biomimetic nanoparticles as an emerging platform for targeted cancer therapy. J Nanobiotechnol. 21(1):287. doi: 10.1186/s12951-023-02064-1. PMC10463632 37608298

[CIT0023] Marinho A, Nunes C, Reis S. 2021. Hyaluronic acid: a key ingredient in the therapy of inflammation. Biomolecules. 11(10):1518. doi: 10.3390/biom11101518. 34680150 PMC8533685

[CIT0024] Marquart J, Chen EY, Prasad V. 2018. Estimation of the percentage of US patients with cancer who benefit from genome-driven oncology. JAMA Oncol. 4(8):1093–1098. doi: 10.1001/jamaoncol.2018.1660. 29710180 PMC6143048

[CIT0025] Mecocci S, Ottaviani A, Razzuoli E, Fiorani P, Pietrucci D, De Ciucis CG, Dei Giudici S, Franzoni G, Chillemi G, Cappelli K. 2022. Cow Milk extracellular vesicle effects on an in vitro model of intestinal inflammation. Biomedicines. 10(3):570. doi: 10.3390/biomedicines10030570. 35327370 PMC8945533

[CIT0026] Mendes TFS, Kluskens LD, Rodrigues LR. 2015. Triple negative breast cancer: nanosolutions for a big challenge. Adv Sci. 2(11):1500053. doi: 10.1002/advs.201500053. PMC5115335 27980912

[CIT0027] Moll J, Khaldoyanidi S, Sleeman J, Achtnich M, Preuss I, Ponta H, Herrlich P. 1998. Two different functions for CD44 proteins in human myelopoiesis. J Clin Invest. 102(5):1024–1034. doi: 10.1172/JCI2494. 9727071 PMC508968

[CIT0028] Munagala R, Aqil F, Jeyabalan J, Agrawal AK, Mudd AM, Kyakulaga AH, Singh IP, Vadhanam MV, Gupta RC. 2017. Exosomal formulation of anthocyanidins against multiple cancer types. Cancer Lett. 393:94–102. doi: 10.1016/j.canlet.2017.02.004. 28202351 PMC5837866

[CIT0029] Munagala R, Aqil F, Jeyabalan J, Gupta RC. 2016. Bovine milk-derived exosomes for drug delivery. Cancer Lett. 371(1):48–61. doi: 10.1016/j.canlet.2015.10.020. 26604130 PMC4706492

[CIT0030] Ong SL, Blenkiron C, Haines S, Acevedo-Fani A, Leite JAS, Zempleni J, Anderson RC, McCann MJ. 2021. Ruminant milk-derived extracellular vesicles: a nutritional and therapeutic opportunity? Nutrients. 13(8):2505. doi: 10.3390/nu13082505. 34444665 PMC8398904

[CIT0031] Paulis YW, Huijbers EJ, van der Schaft DW, Soetekouw PM, Pauwels P, Tjan-Heijnen VC, Griffioen AW. 2015. CD44 enhances tumor aggressiveness by promoting tumor cell plasticity. Oncotarget. 6(23):19634–19646. doi: 10.18632/oncotarget.3839. 26189059 PMC4637310

[CIT0032] Pullan J, Dailey K, Bhallamudi S, Feng L, Alhalhooly L, Froberg J, Osborn J, Sarkar K, Molden T, Sathish V, et al. 2022. Modified bovine milk exosomes for doxorubicin delivery to triple-negative breast cancer cells. ACS Appl Bio Mater. 5(5):2163–2175. doi: 10.1021/acsabm.2c00015. PMC9245909 35417133

[CIT0033] Qhattal HSS, Liu X. 2011. Characterization of CD44-mediated cancer cell uptake and intracellular distribution of hyaluronan-grafted liposomes. Mol Pharm. 8(4):1233–1246. doi: 10.1021/mp2000428. 21696190 PMC3196641

[CIT0034] Rennick JJ, Johnston AP, Parton RG. 2021. Key principles and methods for studying the endocytosis of biological and nanoparticle therapeutics. Nat Nanotechnol. 16(3):266–276. doi: 10.1038/s41565-021-00858-8. 33712737

[CIT0035] Rios de la Rosa JM, Pingrajai P, Pelliccia M, Spadea A, Lallana E, Gennari A, Stratford IJ, Rocchia W, Tirella A, Tirelli N. 2019. Binding and internalization in receptor‐targeted carriers: the complex role of CD44 in the uptake of hyaluronic acid‐based nanoparticles (siRNA delivery). Adv Healthc Mater. 8(24):e1901182. doi: 10.1002/adhm.201901182. 31738017

[CIT0036] Santos-Coquillat A, González MI, Clemente-Moragón A, González-Arjona M, Albaladejo-García V, Peinado H, Muñoz J, Ximénez Embún P, Ibañez B, Oliver E, et al. 2022. Goat milk exosomes as natural nanoparticles for detecting inflammatory processes by optical imaging. Small. 18(6):e2105421. doi: 10.1002/smll.202105421. 34854563

[CIT0037] Sedykh SE, Burkova EE, Purvinsh LV, Klemeshova DA, Ryabchikova EI, Nevinsky GA. 2020. Milk exosomes: isolation, biochemistry, morphology, and perspectives of use. In: Gil De Bona A, Reales-Calderon JA, editors. Extracellular vesicles and their importance in human health. London: IntechOpen. Chapter 2; [accessed 2025 Jun 2].

[CIT0038] Sheridan C, Kishimoto H, Fuchs RK, Mehrotra S, Bhat-Nakshatri P, Turner CH, Goulet R, Badve S, Nakshatri H. 2006. CD44+/CD24-breast cancer cells exhibit enhanced invasive properties: an early step necessary for metastasis. Breast Cancer Res. 8(5):R59. doi: 10.1186/bcr1610. 17062128 PMC1779499

[CIT0039] Soares FA, Salinas B, Reis S, Nunes C. 2023. Milking the milk: exploiting the full potential of milk constituents for nature-derived delivery systems. Trend Food Sci Technol. 141:104209. doi: 10.1016/j.tifs.2023.104209.

[CIT0040] Somiya M, Yoshioka Y, Ochiya T. 2018. Biocompatibility of highly purified bovine milk-derived extracellular vesicles. J Extracell Vesicles. 7(1):1440132. doi: 10.1080/20013078.2018.1440132. 29511463 PMC5827637

[CIT0041] Spadea A, Rios de la Rosa JM, Tirella A, Ashford MB, Williams KJ, Stratford IJ, Tirelli N, Mehibel M. 2019. Evaluating the efficiency of hyaluronic acid for tumor targeting via CD44. Mol Pharm. 16(6):2481–2493. doi: 10.1021/acs.molpharmaceut.9b00083. 31013093

[CIT0042] Sverdlov ED. 2012. Amedeo Avogadro’s cry: what is 1 µg of exosomes? Bioessays. 34(10):873–875. doi: 10.1002/bies.201200045. 22815202

[CIT0043] Vaswani K, Mitchell MD, Holland OJ, Qin Koh Y, Hill RJ, Harb T, Davies PS, Peiris H. 2019. A method for the isolation of exosomes from human and bovine milk. J Nutr Metab. 2019:5764740–5764746. doi: 10.1155/2019/5764740. 31885909 PMC6914892

[CIT0044] Wang L, Shi Z, Wang X, Mu S, Xu X, Shen L, Li P. 2021. Protective effects of bovine milk exosomes against oxidative stress in IEC-6 cells. Eur J Nutr. 60(1):317–327. doi: 10.1007/s00394-020-02242-z. 32328746

[CIT0045] Yang C, He Y, Zhang H, Liu Y, Wang W, Du Y, Gao F. 2015. Selective killing of breast cancer cells expressing activated CD44 using CD44 ligand-coated nanoparticles in vitro and in vivo. Oncotarget. 6(17):15283–15296. doi: 10.18632/oncotarget.3681. 25909172 PMC4558151

[CIT0046] Zhang M, Yang J, Deng F, Guo C, Yang Q, Wu H, Ni Y, Huang L, Chen L, Ding C. 2019. Dual-functionalized hyaluronic acid as a facile modifier to prepare polyanionic collagen. Carbohydr Polym. 215:358–365. doi: 10.1016/j.carbpol.2019.03.086. 30981365

[CIT0047] Zhao S, Chen C, Chang K, Karnad A, Jagirdar J, Kumar AP, Freeman JW. 2016. CD44 expression level and ­isoform contributes to pancreatic cancer cell plasticity, invasiveness, and response to therapy. Clin Cancer Res. 22(22):5592–5604. doi: 10.1158/1078-0432.CCR-15-3115. 27267855 PMC5143222

[CIT0048] Zhong L, Liu Y, Xu L, Li Q, Zhao D, Li Z, Zhang H, Zhang H, Kan Q, Sun J, et al. 2019. Exploring the relationship of hyaluronic acid molecular weight and active targeting efficiency for designing hyaluronic acid-modified nanoparticles. Asian J Pharm Sci. 14(5):521–530. doi: 10.1016/j.ajps.2018.11.002. 32104479 PMC7032078

