# Supplementary material for: Hyaluronic acid-engineered milk extracellular vesicles to target triple negative breast cancer through CD44
Source: Pharm Biol. 2025 Jun 5;63(1):411–27. doi: 10.1080/13880209.2025.2511807 (PMC12143002; doi:10.1080/13880209.2025.2511807)
Supplement: Supplementary_material.pdf [file IPHB_A_2511807_SM3385.pdf]

Supplementary Information

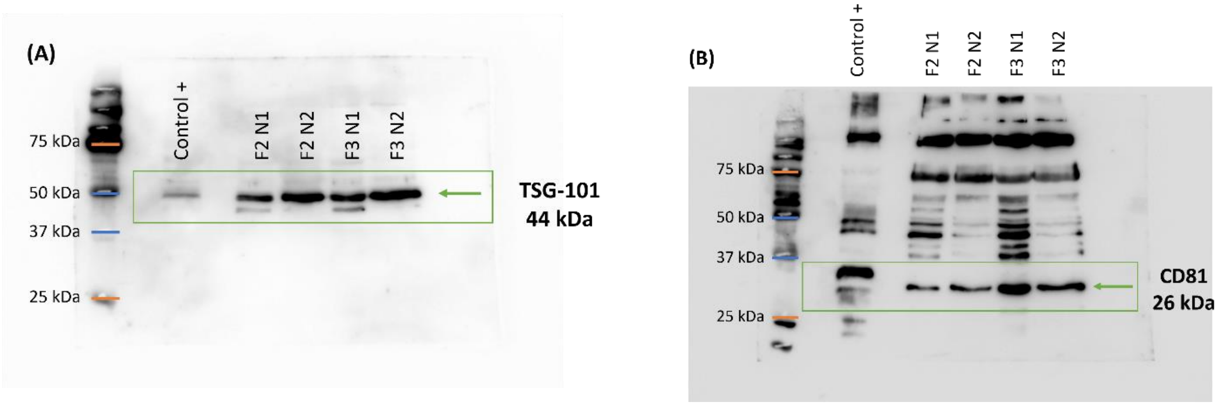

Figure S1. *Qualitative analysis of TSG-101(A) and CD81(B) protein markers presence by western blot, on the collected fractions 2 and 3 (in duplicate, N1 and N2) after the purification of the sEVs by size exclusion chromatography.*

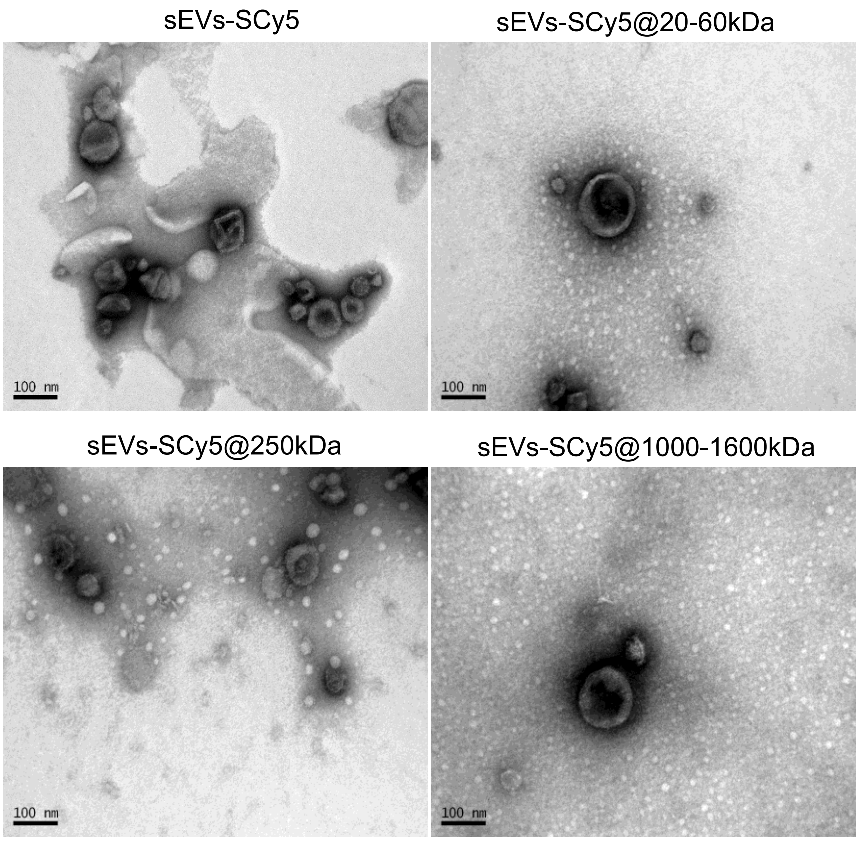

Figure S2. *TEM images for morphological evaluation of the labelled and functionalized sEVs*
